# The Rising Era of Immune Checkpoint Inhibitors in Myelodysplastic Syndromes

**DOI:** 10.1155/2018/2458679

**Published:** 2018-11-01

**Authors:** Nora Chokr, Rima Patel, Kapil Wattamwar, Samer Chokr

**Affiliations:** ^1^Department of Internal Medicine, Yale School of Medicine, New Haven, CT, USA; ^2^Waterbury Hospital, Waterbury, CT, USA; ^3^Tufts Medical Center, Boston, MA, USA; ^4^Medical University of Varna, Varna, Bulgaria

## Abstract

Myelodysplastic syndromes (MDS) are a heterogeneous group of diseases characterized by ineffective hematopoiesis and a wide spectrum of manifestations ranging from indolent and asymptomatic cytopenias to acute myeloid leukemia (AML). MDS result from genetic and epigenetic derangements in clonal cells and their surrounding microenvironments. Studies have shown associations between MDS and other autoimmune diseases. Several immune mechanisms have been identified in MDS, suggesting that immune dysregulation might be at least partially implicated in its pathogenesis. This has led to rigorous investigations on the role of immunomodulatory drugs as potential treatment options. Epigenetic modification via immune check point inhibition, while well established as a treatment method for advanced solid tumors, is a new approach being considered in hematologic malignancies including high risk MDS. Several trials are looking at the efficacy of these agents in MDS, as frontline therapy and in relapse, both as monotherapy and in combination with other drugs. In this review, we explore the utility of immune checkpoint inhibitors in MDS and current research evaluating their efficacy.

## 1. Introduction

Myelodysplastic syndromes (MDS) are a complex set of diseases characterized by ineffective hematopoiesis and a wide spectrum of manifestations, ranging from indolent and asymptomatic cytopenias to acute myeloid leukemia (AML). Most patients are elderly with the vast majority diagnosed after the age of 60 years [1]. According to the World Health Organization (WHO) classification, diagnosis of MDS is still based mostly on histologic and cytologic examination of the bone marrow and peripheral blood. A large number of somatic driver mutations in splicing factors and other epigenetic regulators are thought to have diagnostic and prognostic implications, with the exception of del(5q) and SF3B1 which are mentioned in the classification [2, 3] (Table 1). Patients are risk stratified using several scores including the International Prognostic Scoring System (IPSS), revised IPSS, and the MD Anderson Cancer Center scores. Low risk MDS patients remain stable for years with a 4-year survival rate of 80%, whereas high risk MDS is associated with poor outcomes and rapid progression to leukemia with a median survival of less than a year [2].

Standard of care in MDS includes supportive care with blood transfusions, hematopoietic growth factors, immune modulation with lenalidomide in del(5q), and epigenetic modulation by hypomethylating agents (HMA) such as azacitidine and decitabine. Lenalidomide and HMA have led to a decrease in transfusion dependency and progression to AML. Azacitidine in particular has been associated with an increase in overall survival [4]. However, response to these agents can be lost over time, which emphasizes the importance of gaining a deeper understanding of the disease pathogenesis and development of novel therapies.

## 2. Immune Mechanisms in MDS

Several studies have shown an association between autoimmune diseases and low risk MDS. People with preexisting autoimmune disease have an odds ratio of 1.5 to 3.5 for developing MDS [5–7]. There are several immune based mechanisms identified in MDS, suggesting that immune dysregulation might be at least partially implicated in the pathogenesis of MDS.

**Figure 1:**
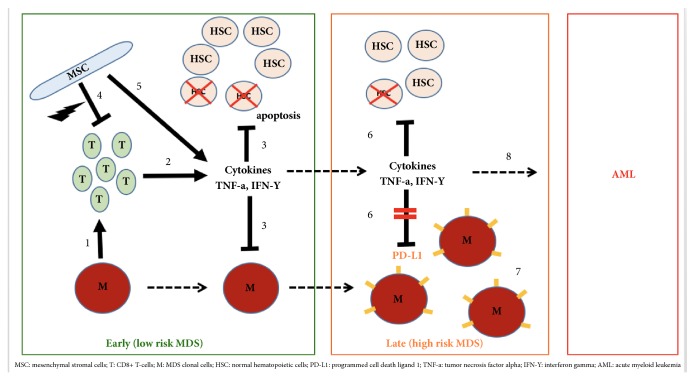
Immune mechanisms in MDS.** Early (low risk) MDS**. (1) Malignant MDS cells (M) induce clonal expansion of CD8+ T-cells (T). (2) CD-8+ T-cells produce cytokines like TNF-a and INF-Y. (3) This results in apoptosis in hematopoietic stem cells (HSC) resulting in cytopenias but also keep MDS cells (M) from proliferating. (4) MSC in normal hematopoiesis suppress T-cell activation, a process that is aberrant in MDS. (5) MSC also produce proinflammatory cytokines.** Late (high risk) MDS**. (6) While continued inflammation leads to more apoptosis of hematopoietic stem cells (HSC) and cytopenias worsen, TNF-a and INF-Y start inducing PDL-1 expression on MDS clonal cells. (7) PD-L1 allows the MDS cells to escape immune surveillance by T-cell suppression.** AML.** (8) MDS transitions into acute myeloid leukemia (AML).

At the level of the bone marrow, normal hematopoiesis is regulated by the immune system via a complex interplay between T-cells, cytokines, innate immunity, and mesenchymal stromal cells (MSC) Figure 1. In MDS, immune dysregulation occurs through several mechanisms including T-cell mediated bone marrow suppression, expression of cytokines, overactivation of pathways involved in innate immunity, and altered mesenchymal stromal cells (MSC). We elaborate on each of these mechanisms to better understand the pathogenesis of MDS.

In low risk MDS, apoptosis plays a key role through T-cell inhibition. Many MDS patients have oligoclonal T-cells, largely derived from a malignant MDS clone. These cytotoxic CD8+ T-cells recognize MHC-class I molecules on progenitor cells inhibiting growth and leading to cytopenias and abnormal hematopoiesis. These cytotoxic T-cells carry specific TCR subtypes that intoxicate hematopoietic cells [8, 9]. A study by Wu et al. showed the possible inhibitory function of T-cells was likely due to type-1 polarization involving both the CD-4+ and CD-8+ subsets [10].

Another study has questioned the autoreactivity of T-cells in MDS which was suggested by a defective in vitro cytotoxicity [11]. Overall, studies have shown that cytotoxic T-cells are likely involved in autoimmune reactions towards hematopoietic cells. These immune mechanisms have been further reinforced in trials that showed hematopoietic recovery and delayed progression to AML with immunosuppressive therapies that block the cytotoxic effects of CD8+ lymphocytes [8, 9].

Several cytokines are abnormally expressed in MDS, either by clonal cells or surrounding stromal cells. Tumor necrosis factor alpha (TNF-*α*) and interferon gamma (IFN-*γ*) are overly expressed in MDS [12–14]. These cytokines increase the expression of Fas receptors (FasR) on hematopoietic cells and enhance their interaction with Fas ligands leading to apoptosis [15]. TNF-*α* and IFN-*γ* were shown to induce the immunoinhibitory molecule B7-H1, via nuclear factor-kappa B activation in blasts of MDS patients [16]. The role of TGF- *β* cytokine in inhibition of normal stem cells is also well established, and its pathway has been recently targeted by several drugs. TGF-*β* binds to a set of TGF-*β* receptors and leads to the activation of intracellular SMAD 2/3 proteins [12–15]. The levels of TNF-*α* and TGF-B are inversely related to hemoglobin and survival [8]. These cytokines also induce the expression of programmed death ligand 1 (PD-L1) on tumor cells, a mechanism that can potentially allow tumor cells to escape from the immune mediated tumor surveillance. CD3+ CD4+ interleukin (IL)-17 producing T-cells have been shown to be upregulated in low risk MDS, and higher levels have been also associated with more severe anemia [17, 18].

Myeloid-derived suppressor cells (MDSC) were shown to be increased in the bone marrow of MDS patients. These cells overproduce cytokines that suppress normal hematopoiesis and induce mechanisms that target hematopoietic progenitors leading to increased apoptosis. The expansion of MDSC results from the interaction of the proinflammatory molecule S100A9 with CD33 and the subsequent production of the proinflammatory interleukin-10 and TGF-B [19, 20].

Innate immunity also plays a role in MDS. Innate immunity depends on pattern recognition of microbial markers by receptors such as toll-like receptors (TLRs). TLR-2 and TLR-4 are upregulated in the bone marrow of MDS patients. TLR-4 expression is correlated with increased apoptosis [21]. Overactive TLRs lead to overexpression of activators such as MYD88, TIRAP, IRAK1/4, and TRAF and downregulation of inhibitory factors such as miR145 and miR146a. This subsequently enhances the NF-kB and mitogen-activated protein kinase (MAPK) pathways and ultimately increases the production of inflammatory cytokines [22–24]. Interestingly, MYD88 blockade leads to an increase in erythroid colony formation [25].

MDS is characterized by an inefficient dendritic cells (DC) pool likely from the decreased ability of monocytes to differentiate fully into mature DC. DC derived in vitro from peripheral blood mononuclear cells of MDS patients were reduced in numbers compared with healthy controls. DC in MDS express lower levels of CD1a, CD54, CD80, and MHC II molecules [26]. Immature DC have an impaired cytokine secretion which likely accounts for their reduced allostimulatory capacity [27].

Normal hematopoiesis is a fine balance that depends not only on the hematopoietic progenitor cells, but also on the surrounding MSC. They play a pivotal role in the birth of MDS clones and other myeloid malignancies. In MDS, MSC may be absent or dysfunctional due to genetic aberrations. The selective deletion of Dicer1 gene in MSC cells of murine models was shown to induce MDS and AML [28]. Research has shown that cytogenetically abnormal MSC in MDS lead to the production of proinflammatory cytokines such as TNF-*α*, IL-6, TGF-B, and IFN-*γ* [29, 30]. Normally, MSC exert immunosuppressive effects on the surrounding T-cells through paracrine and cell-to-cell interactions, which then arrests T-cells in the G1-phase and diminishes their cytokine secretion [8, 31]. However, this immunosuppressive effect on CD 8+ T-cells can become aberrant in MDS. Interestingly, significant differences in the immunoregulatory functions were demonstrated between MSC in low risk MDS versus high risk MDS. In high risk MDS, MSC are characterized by increased TGF-B1 expression, apoptosis, immunosuppressive rate, and reduced hematopoietic support ability [31]. MSC in low risk MDS are also characterized by a poor ability to suppress dendritic cells differentiation and maturation [32].

Though these intricate immune mechanisms suggest that immunomodulatory and immunosuppressive therapies can be potential treatment options for MDS, these therapies were interestingly trialed even before the underlying mechanisms were completely understood. Immunosuppressive drugs such as antithymocyte globulin (ATG), cyclosporine (CS), and mycophenolate mofetil (MM) have been studied in low risk MDS [33], but still underutilized in MDS patients due to conflicting data and variable rates of response reported by the different studies [34–36]. Also, MAPK and TLR inhibitors, which target overactive TLR pathways in MDS, are currently being evaluated in several clinical trials [8, 37, 38]. Checkpoint inhibitors are another family of drugs that have been approved for solid tumors and now being investigated in high risk MDS and other hematologic malignancies such as AML [1].

## 3. The Rise of Immune Checkpoint Inhibitors

The concept of developing therapies targeting the immune system rather than tumor cells originated from Dr. James Allison's discovery of the cytotoxic T-lymphocyte antigen 4 (CTLA-4), a T-cell receptor that downregulates T-cells and the immune system. This led to the development of ipilimumab (Yervoy, Bristol-Myers Squibb), a human IgG1 CTLA-4 checkpoint inhibitor which blocks T-cell suppression and upregulates antitumor immune defenses [39]. Programmed cell death receptor (PD-1) and programmed cell death ligand (PD-L1) represent an immune checkpoint involved in regulating T-cells at the level of the peripheral tissues. Tumors can express PD-L1 and use these ligands to evade the host's immune system, making this checkpoint a potential target for cancer therapy [40, 41]. This pathway inspired the development of monoclonal antibodies that block the interaction between PD-1 receptors and PD-L1 ligands to help restore anticancer immune responses [42]. These agents have proven to be very effective in tumors refractory to standard chemotherapy regimens in more than 10 organ systems.

Pembrolizumab (Keytruda, Merck) was the first PD1 inhibitor approved by the US Food and Drug Administration (FDA) in September 2014 for treatment of advanced melanoma refractory to BRAF inhibitors and ipilimumab. Nivolumab (Opdivo, Bristol-Myers Squibb), the fully human IgG4 anti-PD-1 antibody, was approved by the FDA in December 2014 for unresectable or metastatic melanoma that progressed after ipilimumab therapy and for patients with positive* BRAF* V600 mutation who failed treatment with BRAF inhibitors. The approval came after the landmark clinical trial Checkmate-037 [43]. In addition to melanoma, Nivolumab is now FDA approved for metastatic non-small cell lung cancer, metastatic renal cell carcinoma and urothelial cancers, refractory Hodgkin's lymphoma, cancers of the head and neck, and hepatocellular carcinoma. Results in non-Hodgkin's lymphoma were favorable in refractory follicular lymphoma. Results in relapsed diffuse large B-cell lymphoma following autologous stem cell transplantation have shown an overall survival probability of 82% at 16 months following treatment with pidilizumab, an experimental PD-1 inhibitor. Monotherapy with anti-PD-1 agents in multiple myeloma has not shown any promising results; however, current trials are studying the effect of anti-PD-1 agents in combination with standard myeloma therapy. Preliminary data from these trials has shown a synergistic effect and higher response rates compared to standard chemotherapy alone [39, 44, 45]. There are several ongoing clinical trials investigating the role of PD-1 blockage in myelodysplastic syndromes as well, either as monotherapy or in combination with other therapies.

The use of checkpoint inhibitors is expected to rise dramatically as we learn more about their efficacy across a wide range of cancers. These medications, however, are not harmless. Some of the adverse effects are mild and easily controlled with systemic steroids, but others can be serious and fatal. In their attempt to augment the immune response, PD-1 inhibitors can breach immunologic tolerance by upregulating autoreactive T-cells causing immune mediated reactions such as rash, pneumonitis, colitis, thyroiditis, hepatitis, nephritis, uveitis, adrenalitis, facial nerve paresis, hypophysitis, aseptic meningitis, and fulminant diabetes [41]. The role of autoimmunity has been recognized as a pathogenic mechanism in low risk MDS but is still under discussion and not clearly delineated. In high risk MDS, the role of autoimmunity is even less so understood. Check point inhibitors should be used with caution in high risk MDS due to their potential autoimmune side effects.

## 4. MDS and Checkpoint Inhibitors

Epigenetic modification via PD-1 inhibition is a new approach being considered in high risk MDS and other hematologic malignancies. Yang et al. [46] reported abnormal upregulation of PD-L1, PD-L2, PD-1, and CTLA4 in CD34+ cells in MDS patients compared to healthy controls. This may be one of the major mechanisms triggering high risk MDS. Patients with high risk MDS have a higher percentage of PD-L1 expression on their blasts compared to those with low risk MDS. This upregulation of PD-1/PD-L1 is evident not only in the clonal cells but also in their surrounding mesenchymal cells. Dail et al. [47] measured PD-L1 expression using multicolor flow cytometry and immunohistochemistry and found that PD-L1 is detectable in more than 2% of cells in all MDS patients, with the majority of expression occurring in non-tumor hematopoietic cells. This supports that the upregulation of PD-1/PD-L1 occurs in both clonal cells and MSC. The interaction between these cells and PD-1/PD-L1 leads to T-cell suppression and influences cell cycle progression. This also results in genetic and epigenetic modulation leading to increased apoptosis and cancer tolerance [48]. Some of the mechanisms implicated in this T-cell suppression include inhibition of Lck/ZAP-70 and PI3K-Akt/MEK-ERK MAPK pathways, indirect activation of p38 MAPK, increased proliferation of T regulatory cells with suppressor effects on the immune response, and inhibition of T-cell receptor mediated lymphocyte proliferation and cytokine secretion [2].

There are some trials evaluating the role of checkpoint inhibitors as monotherapy in MDS. Garcia-Manero et al. conducted a phase Ib trial to investigate the efficacy and side effect profile of the PD-1 inhibitor, pembrolizumab, in 28 MDS patients with intermediate 1 or 2 or high risk MDS who failed therapy with HMA. The trial showed favorable outcomes. Efficacy was evaluated in 27 patients using the IWG 2006 response criteria for MDS [49]. Partial response was observed in 1 patient (3%), complete marrow response in 3 patients (11%), and stable disease in 14 patients (52%) and progressive disease was noted in 9 patients (33%). The median overall survival for all patients was 23 weeks, and 4 of the 27 patients were alive for more than 2 years [50]. Adverse effects were noted in 10 of 28 patients with most common effects being fatigue and hypothyroidism. There was one case of grade 3 gastroenteritis and one case of grade 4 tumor lysis syndrome [1].

The CTLA-4 inhibitor, ipilimumab, yielded stabilization of high risk MDS in 5 of 11 patients in a phase Ib study [51]. Zeidan et al. [51] studied the efficacy of ipilimumab in high risk MDS patients who failed HMA in a multicenter phase Ib clinical trial enrolling 29 patients with IPSS intermediate-1 or 2 to high risk. Two complete marrow responses were reported (7%). Prolonged stable disease for 46 weeks or more occurred in 6 patients and for 54 weeks or more in 3 patients. Median overall survival was 294 days and for those who received maintenance ipilimumab, it was 400 days. Patients who responded had increased expression of inducible T-cell costimulator (ICOS), a biomarker of T-cell activation. This trial revealed that the effect of ipilimumab monotherapy while effective in some cases is, however, limited and more combination-based approaches should be considered for more significant response rates.

Since research on checkpoint inhibitors as monotherapy in HMA refractory patients has shown limited response, checkpoint inhibitors are now being studied in combination with other MDS therapies particularly HMA to assess for synergistic response. Wrangle et al. [52] were the first to describe the sensitization effect of HMA to anti-PD-1 agents. They noticed high response rates in non-small cell lung cancer patients who failed treatment with azacitidine and were later enrolled in a trial with a PD-1 inhibitor. HMA were found to induce viral mimicry by upregulating endogenous retroviruses (ERVs) leading to upregulation of checkpoint pathways, increase in apoptosis, and sensitization of MDS cells to checkpoint inhibitors [53–55]. HMA increase PD-1 expression in peripheral blood mononuclear cells and bone marrow cells [46]. If resistance to HMA is at least partially mediated by the increased activation of checkpoint pathways, then combining both HMA and checkpoint inhibitors should theoretically yield more favorable results. Another mechanism by which HMA increase the sensitivity to PD-1/PD-L1 inhibition is through the suppression of myeloid-derived suppressor cells (MDSCs) in the bone marrow [48]. While HMA are thought to increase sensitivity to PD-1 inhibitors by upregulating PD-1, PD-L1, and PD-L2, it remains unclear if blocking the PD-1/PD-L1 pathway will result in restoring sensitivity to HMA in treatment refractory patients. Garcia-Manero et al. evaluated multiple cohorts that included cohort 1 treated with nivolumab alone, cohort 2 with ipilimumab alone, and cohort 3 with nivolumab combined with ipilimumab. All three cohorts evaluated MDS patients who progressed on HMA therapy. The study also included cohort 4 treated with azacitidine and nivolumab, cohort 5 azacitidine combined with ipilimumab, and cohort 6 azacitidine combined with nivolumab and ipilimumab, all of which were previously untreated intermediate 2 to high risk MDS patients. Cohort 1 that received nivolumab alone did not show any significant response. Cohort 2 that received ipilimumab showed an overall response rate of 30% (5 of 16) with 18% of the patients (3 of 16) experiencing grade 3 or more adverse events related to the therapy [56]. The response rate in cohort 4 that received a combination of azacitidine and nivolumab was 80% (13 of 17). It is noteworthy that in cohort 1, patients received nivolumab alone after failing treatment with HMA and did not have any significant response, whereas the cohort receiving combination therapy with nivolumab and azacitidine had a good response rate, suggesting that both drugs are likely working synergistically. The higher response rate of azacitidine and nivolumab suggest that combination therapy is more effective than nivolumab monotherapy [1]. Data from cohort 6 is not yet available (Table 2).

PD-L1 inhibitors atezolizumab and durvalumab are currently being investigated alone and in combination with HMA [39]. Other ongoing trials in MDS include pembrolizumab in combination with entinostat, a histone deacetylase inhibitor, in HMA refractory patients. Two ongoing phase 1b trials are studying atezolizumab in combination with other agents in previously untreated patients (Table 2).

## 5. Conclusion

MDS are a complex disease resulting from genetic and epigenetic derangements in clonal cells and their surrounding microenvironment. Over the past 10 years, our deeper understanding of the disease has allowed for great pharmacologic and therapeutic advancements with azacitidine, decitabine, and lenalidomide. Unfortunately, the results are still not optimal. It is not surprising given the involvement of several signaling pathways and underlying mechanisms as patients often do not respond or lose response over time to a specific therapy. There is still a significant knowledge gap that needs to be filled for MDS. Several somatic mutations have been identified in MDS suggesting that precision medicine with targeting of actionable mutations such as IDH1/2, RAS, JAK, and FLT3 may be an attractive approach [57]. One should keep in mind that targeting one specific mutation may only be a short-term solution as clonal cells may develop new escape mechanisms and novel mutations leading to loss of response. We are in need of therapeutic options that can be used in MDS regardless of genomic profiles. Immunotherapy is a new approach that is independent of genomic background [57]. It is unclear whether immune checkpoint inhibitors will provide any benefit as monotherapy. Preliminary data from clinical trials show promising results when these agents are combined with HMA in patients who initially failed HMA monotherapy, suggesting synergistic effect between the drugs. Currently, there are some phase I trials studying the efficacy of checkpoint inhibitors in patients with MDS, both as frontline therapy and in refractory disease [57].

Finally, as we gain a better understanding of the intricate pathophysiologic basis of MDS, it is not unreasonable to assume that combining several agents together, each acting via a different mechanism, is likely to achieve more promising results, albeit at the expense of potentially more significant drug related adverse events. This approach will likely be the main focus of future research. It is challenging to predict outcomes to therapy in MDS as they may vary with the specific population. Efforts should be directed to elicit specific characteristics to help predict response in selected populations.

## Figures and Tables

**Table 1:** Common gene mutations in MDS and the prognostic values [3].

**Genes**	**Prognostic Impact**
**Epigenetic Regulators**	
TET2	poor
EZH2	poor
ASXL1	poor
DNMT3A	poor
IDH1/IDH2	poor
**Spliceosomal genes**	
SF3B1	good
U2AF1	poor
SRSF2	poor
ZRSR2	unknown
**Cytoplasmic tyrosine kinases **	
JAK2	poor
**Signaling molecules **	
SETBP1	poor
NRAS	poor
**Transcriptional factors**	
ETV6	poor
RUNX1	poor
**Tumor suppressors**	
TP53	poor
ROBO1/ROBO2	poor
**Chromatid cohesion**	
STAG2	poor

**Table 2:** Ongoing and future monotherapy and combination phase I/II trials with immune check point inhibitors in MDS and interim results.

**Trials**	**Authors**	**Treatment Arms**	**Estimated MDS Participants**	**Inclusion Criteria**	**Outcomes**	**Comments**
**A Phase I Trial of Ipilimumab in Patients with Myelodysplastic Syndromes after Hypomethylating Agent Failure (NCT01757639)**	Zeidan et al	Ipilimumab alone	29	HMA failure^*∗*^or HMA refusal IPSS int-1 with excess BM blasts ≥ 5% or transfusion dependency, Int-2 or high risk;	mCR 7%; PSD 31%; OS 294 days and 400 days with maintenance	Phase 1

**Stabilization of Myelodysplastic Syndromes (MDS) Following Hypomethylating Agent (HMAs) Failure Using the Immune Checkpoint Inhibitor Ipilimumab: A Phase I Trial**	Zeidan et al	Ipilimumab alone	11	Primary or secondary failure of HMAs^*∗*^ IPSS int-1 with excess BM blasts ≥ 5% or transfusion dependency, Int-2 or high risk;	SD >6 m 27.3%; SD >16 m 9%; PSD 9%; median OS 368 d; mean OS 352 d; 3 patients underwent alloSCT and remained in CR at 2,12,18 m post SCT	Showed that 3 mg/kg dose is tolerable and can lead to prolonged disease stabilization; alloSCT is feasible post ipilimumab phase 1

**A Trial of Pembrolizumab (MK-3475) in Participants With Blood Cancers (MK-3475-013/KEYNOTE-013) (NCT01953692)**	Garcia-Manero et al	Pembrolizumab alone	28	HMA failure IPSS int-1,int-2, or high risk	mCR 11%; PR 3%; SD 52%; HI 11%; PD 33%; median OS 23 weeks; 15% alive for >2 years	Phase 1

**Nivolumab and Ipilimumab With 5-azacitidine in Patients With Myelodysplastic Syndromes (MDS) (NCT02530463)**	Garcia-Manero et al	Cohort 1 nivolumab alone; Cohort 2 ipilimumab alone; Cohort 3 nivolumab + ipilimumab; Cohort 4 azacitidine + nivolumab; Cohort 5 azacitidine + ipilimumab; Cohort 6 azacitidine + nivolumab + ipilimumab	120	Cohorts 1,2,3 in HMAs failure^*∗∗*^ in any IPSS risk; Cohorts 4,5,6 frontline therapy in int-2/high risk MDS	**Limited data available** **Cohort 1: **No response noted; **Cohort 2**: ORR 30% (includes CR in 1, mCR in 2, HI in 2) in int/high-risk MDS, NR 56%, PD 12%; **Cohort 4: **ORR 80% (CR 6 of 17, mCR+HI in 6 of 17, HI-P in 1), PD 12%, Was too early to evaluate 2 pts	Data from cohorts 1,2,4 was presented at ASH 2016 phase 2

**Lirilumab and Nivolumab With 5-Azacitidine in Patients With Myelodysplastic Syndromes (NCT02599649)**	Garcia Manero et al	Cohort 1 lirilumab alone; Cohort 2: nivolumab + lirilumab; Cohort 3: azacitidine + lirilumab; Cohort 4: azacitidine + lirilumab + nivolumab	12 participants	HMA Naïve Cohort 1, 2 low risk/int-1 MDS Cohort 3,4 high int-2/risk MDS	No data available yet	Cohort 1, 2 could have received prior non-methylating agents. No prior anti-PD-1 or PD-L1 or immune activating drugs. Prior anti-CTLA-4 is allowed as long as the last dose was >101 days ago. Phase 2

**An Efficacy and Safety Study of Azacitidine Subcutaneous in Combination With Durvalumab in Previously Untreated Subjects With Higher-Risk Myelodysplastic Syndromes (NCT02775903)**	NA	Randomizes participants to either azacitidine alone OR azacitidine + durvalumab	Target MDS participants: 72	Frontline treatment in IPSS-R intermediate >10% blasts or poor or very poor cytogenetics; OR IPSS-R High or very high risk	No data available yet	Enrollment began in June 2016. Phase 2

**Entinostat and Pembrolizumab in Treating Patients With Myelodysplastic Syndrome After DNMTi Therapy Failure (NCT02936752)**	Zeidan et al	Entinostat + Pembrolizumab	Target MDS participants: 27	DNMTi failure (no CR, PT, HI after at least 4 cycles or progression of disease) regardless of initial IPSS-R	No data available yet	Single arm open label study excludes people who received anti-PD1 or PD-L1 or HDACi or anti-CTLA-4 or other immune activating therapy within the last 3 months

**A Study of Atezolizumab Administered Alone or in Combination With Azacitidine in Participants With Myelodysplastic Syndromes (NCT02508870)**	NA	**HMA R/R MDS**: atezolizumab alone OR atezolizumab + azacitidine **HMA-naïve MDS**: atezolizumab + azacitidine	Target MDS participants: 100	**R/R HMA:** progression at any time on HMA OR no CR or PR or HI after HMA **HMA naïve**: frontline therapy in IPSS-R int, high or very high	No data available yet	Phase 1

**Phase 1 Study to Evaluate MEDI4736 in Subjects With Myelodysplastic Syndrome (NCT02117219)**	NA	MEDI4736 (Durvalumab) alone OR MEDI4236 + azacitidine OR MEDI4736 + tremelimumab OR MEDI4736 + azacitidine + tremelimumab	Target MDS participants: 73	R/R to HMAs or couldn't tolerate HMAs	No data available yet	Phase 1

**Study of PDR001 and/or MBG453 in Combination With Decitabine in Patients With AML or High Risk MDS (NCT03066648)**	NA	Decitabine + PDR001 (spartalizumab) OR Decitabine + MBG453^❊^ OR Decitabine + MBG453 + PDR001	Target MDS participants: 70	HMA Naïve High risk MDS	No data available yet	Prior anti-PD-1/PD-L1 exposure as long as tolerated, or adverse events adequately treated with steroids >7 days of the first dose of study drug are not excluded

HMA: hypomethylating agents; R/R: relapsed/refractory; BM: bone marrow; IPSS-R: revised international prognostic scoring system; MDS: myelodysplastic syndrome; mCR: complete marrow response; PR: partial response; PD: disease progression; HI: hematologic improvement; SD: stable disease; PSD: prolonged stable disease; alloSCT: allogeneic stem cell transplant; HDACi: histone deacetylase inhibitors; NA: not available; PD-1: programmed cell death-1; PD-L1: programmed cell death ligand 1; CTLA-4: cytotoxic T cell associated protein 4; int-1: intermediate 1; int-2: intermediate 2.

^*∗*^HMA failure: failing to achieve CR, PR, or HI after at least 4 cycles.

^*∗∗*^HMA failure defined as relapse or progression after any number of cycles or no response after at least 6 HMA cycles.

^❊^MBG453: T cell immunoglobulin domain and mucin domain 3 (TIM-3) blocker.
